# Stage‐based approach to predict left ventricular reverse remodeling after mitral repair

**DOI:** 10.1002/clc.23879

**Published:** 2022-06-24

**Authors:** Makoto Hibino, Nitish K. Dhingra, Vincent Chan, C. David Mazer, Hwee Teoh, Adrian Quan, Raj Verma, Howard Leong‐Poi, Gianluigi Bisleri, Kim A. Connelly, Subodh Verma

**Affiliations:** ^1^ Division of Cardiac Surgery Li Ka Shing Knowledge Institute of St. Michael's Hospital Toronto Ontario Canada; ^2^ Department of Surgery University of Toronto Toronto Ontario Canada; ^3^ Division of Cardiac Surgery University of Ottawa Heart Institute Ottawa Ontario Canada; ^4^ School of Epidemiology, Public Health and Preventive Medicine University of Ottawa Ottawa Ontario Canada; ^5^ Department of Anesthesia Li Ka Shing Knowledge Institute of St. Michael's Hospital Toronto Ontario Canada; ^6^ Department of Anesthesiology and Pain Medicine University of Toronto Toronto Ontario Canada; ^7^ Department of Physiology University of Toronto Toronto Ontario Canada; ^8^ Division of Cardiac Surgery Li Ka Shing Knowledge Institute of St. Michael's Hospital Toronto Ontario Canada; ^9^ Division of Endocrinology and Metabolism Li Ka Shing Knowledge Institute of St. Michael's Hospital Toronto Ontario Canada; ^10^ Division of Cardiac Surgery Li Ka Shing Knowledge Institute of St. Michael's Hospital Toronto Ontario Canada; ^11^ Royal College of Surgeon Ireland Dublin Ireland; ^12^ Division of Cardiology Li Ka Shing Knowledge Institute of St. Michael's Hospital Toronto Ontario Canada; ^13^ Department of Medicine University of Toronto Toronto Ontario Canada; ^14^ Division of Pharmacology and Toxicology University of Toronto Toronto Ontario Canada

**Keywords:** mitral repair, mitral valve

## Abstract

**Background:**

Although predictors of reverse left ventricular (LV) remodeling postmitral valve repair are critical for guiding perioperative decision‐making, there remains a paucity of randomized, prospective data to support the criteria that potential predictor variables must meet.

**Methods and Results:**

The CAMRA CardioLink‐2 randomized trial allocated 104 patients to either leaflet resection or preservation strategies for mitral repair. The correlation of indexed left ventricular end‐systolic volume (LVESVI), indexed left ventricular end‐diastolic volume (LVEDVI), and left ventricular ejection fraction (LVEF) were tested with univariate analysis and subsequently with multivariate analysis to determine independent predictors of reverse remodeling at discharge and at 12 months postoperatively. At discharge, both LVESVI and LVEDVI were independently associated with their preoperative values (*p* < .001 for both) and LVEF by preoperative LVESVI (*p* < .001). Mitral ring size was favorably associated with the change in LVESVI (*p* < .05) and LVEF (*p* < .01) from predischarge to 12 months, while the mean mitral valve gradient after repair was adversely associated with the change in LVESVI (*p* < .05) and LVEDVI (*p* < .05). No significant associations were found between reverse remodeling and coaptation height nor mitral repair technique.

**Conclusions:**

Beyond confirming the lack of impact of mitral repair technique on reverse remodeling, this investigation suggests that recommending surgery before significant LV dilatation or dysfunction, as well as higher postoperative mitral valve hemodynamic performance, may enhance remodeling capacity following mitral repair.

## 1 INTRODUCTION

Mitral valve repair remains the gold‐standard treatment modality for patients with primary, degenerative mitral regurgitation (MR).^1^ By restoring valve competency, and thus resolving MR‐associated volume overload, the procedure may in turn trigger cardiac reverse remodeling with notable improvements in left ventricular (LV) volumes,^2^, ^3^, ^4^, ^5^, ^6^, ^7^, ^8^, ^9^, ^10^, ^11^, ^12^, ^13^, ^14^ dimensions,^2^, ^4^, ^7^, ^8^, ^10^, ^12^, ^13^, ^15^, ^16^, ^17^, ^18^, ^19^, ^20^, ^21^, ^22^ mass^3^, ^4^, ^9^, ^19^, ^20^, ^23^ and geometry^2^, ^5^, ^23^ postoperatively. Our recent sub‐analysis of the CAMRA CardioLink‐2 randomized trial data confirmed that the reversal of systolic and diastolic indices of cardiac remodeling occur in distinct phases, while also establishing that mitral valve repair technique did not significantly impact the course of this process.

Despite the clear benefits offered by mitral valve repair, the reverse remodeling process can be incomplete in some patients, often manifesting as an inability for LV function to return to baseline levels or long‐term LV hypertrophy.^4^, ^10^, ^11^, ^14^, ^18^, ^19^, ^22^, ^24^ Importantly, postoperative LV dysfunction has been associated with a higher risk for poor clinical outcomes that include all‐cause mortality, cardiac death, cardiac failure, and other cardiac events,^17^, ^22^ and reverse remodeling has been associated with lower symptom burdens, as well as less late atrial fibrillation and cardiac adverse events.^23^ Accordingly, the identification of factors that have the potential to accurately predict which patients are at risk for poor reverse remodeling may help guide perioperative decisions and improve surgical outcomes. Despite laudable attempts to identify potential prognostic factors, randomized, prospective data to help inform the prediction of reverse remodeling amongst patients with primary, degenerative MR remains scarce. Given that the conduct of regular serial echocardiograms following mitral repair may not be a routine practice in certain institutions, the widely utilized retrospective techniques for such analyses are prone to selection bias and are therefore limited in their clinical utility. The purpose of the present investigation was, therefore, to use data from the randomized CAMRA CardioLink‐2 trial, which found no difference in functional mitral stenosis at 12 months amongst patients randomized to leaflet preservation or resection techniques,^25^ to identify factors associated with both immediate and midterm postoperative reverse remodeling following mitral valve repair.

## 2 PATIENTS AND METHODS

### 2.1 Study population

The design and primary results of the CAMRA CardioLink‐2 trial have been reported (Clinicaltrials.gov identifier NCT02552771).^25^, ^26^ In brief, CAMRA CardioLink‐2 was a double‐armed, prospective trial that was conducted at seven specialized Canadian sites which randomized patients with posterior mitral valve prolapse to either leaflet resection or leaflet preservation mitral valve repair strategies. Major exclusion criteria included: anterior leaflet or commissural prolapse, rheumatic mitral valve disease, endocarditis, LV ejection fraction (LVEF) <40%, extensive mitral annular calcification, inability to participate in bicycle ergometry, and planned concurrent aortic valve surgery. Ethics approval was obtained from the individual institutions and informed consent was received from all participants. The study protocol was approved by IRB as REB# 15‐162.

### 2.2 Surgical technique

Intraoperative assessment of the mitral valve to ensure the feasibility and safety of either mitral repair strategy was required before 1:1 randomization to either the leaflet resection or leaflet preservation procedure. More exhaustive accounts of the relevant randomization and surgical processes have been published^25^, ^26^; in brief, triangular or quadrangular resection, with or without simultaneous sliding plasty, was employed in the leaflet resection group while CV4 or CV5 polytetrafluoroethylene sutures stationed on the anterolateral or posteromedial papillary muscle head were utilized in the leaflet preservation group. A volume of at least 15 mitral valve repairs per year and a success rate of greater than 90% were required of all participating surgeons.

### 2.3 Echocardiographic assessment

Echocardiographic assessments were conducted at the baseline visit, predischarge and 12 months postsurgery at an independent core lab and completed in a blinded fashion under the supervision of a level 3 echocardiographer with 20+ years of relevant experience (HLP). The echocardiographic database from the CAMRA CardioLink‐2 trial has been previously published.^25^

### 2.4 Statistical methods

Normality of continuous variables was tested with the Skewness and Kurtosis test. Continuous variables are reported as mean ± *SD*, while frequencies and percentages are used to describe categorical data. To compare multiple time points, we used the repeated measures analysis of variance, followed by paired the Student *t* tests. Linear regressions were used to predict predischarge indexed LV end‐systolic volume (LVESVI), indexed LV end‐diastolic volume (LVEDVI), and LVEF with perioperative, variables. The regressions were also used to predict changes in LVESVI, LVEDVI and LVEF from predischarge to 12 months. The variables tested in univariate analyses included demographic, laboratory, comorbid, echocardiographic, and intraoperative structural variables; those that reached statistical significance for association in univariate regression were included in the multivariate regressions. When analyses yielded significant associations of a particular outcome with both LVESVI and LVEDVI, LVESVI was selected as the relevant predictor for LVESVI and LVEF, while LVEDVI was used to predict LVEDVI, considering their physiological relationship. Furthermore, when cardiopulmonary bypass perfusion time and clamp time were both significant, perfusion time was used for the regression analyses as it had a stronger association with outcomes, as demonstrated by the lower corresponding *p* value. A *p* < .05 was considered statistically significant. All statistical analyses were performed using the STATA statistical software version 14.1 (StataCorp LP).

## 3 RESULTS

### 3.1 Baseline and operative characteristics

A total of 104 patients were randomized in the CAMRA CardioLink‐2 trial, 54 of which were allocated to the leaflet resection group and 50 of which were allocated to the leaflet preservation group. As outlined in Table 1, there was a comparable distribution of baseline and operative characteristics between the two study groups. There were two incidents of investigator‐initiated discontinuations in each group; there were no conversions to mitral valve replacement and no in‐hospital mortality resulting in a total of 52 patients in the resection group and 48 patients in the preservation group at discharge. There were, however, three crossovers from the preservation group and one crossover from the resection group. Data from 46 patients in the resection group and 42 patients in the preservation group were available at the final follow‐up visit for analysis. One patient and two from the resection and preservation groups, respectively, showed evidence of moderate MR before discharge; and one patient from the resection group and two from the preservation group similarly demonstrated moderate MR at 12 months postoperatively.^25^ Baseline echocardiographic values for the entire analyzed cohort are presented in Table S1.

**Table 1:** Baseline characteristics of included patients

	Leaflet resection (*n* = 54)	Leaflet preservation (*n* = 50)
Age at surgery, year	63.9 ± 10.4	66.3 ± 10.8
Women, *n* (%)	10 (18.5)	8 (16.0)
BSA, m^2^	1.9 ± 0.2	1.9 ± 0.2
BMI, kg/m^2^	26.7 ± 3.6	26.4 ± 3.7
Smoking history, *n* (%)	22 (40.7)	16 (32.7)
Hypertension, *n* (%)	22 (40.7)	30 (60.0)
Hyperlipidemia, *n* (%)	24 (44.4)	21 (42.0)
Diabetes mellitus, *n* (%)	2 (3.7)	3 (6.0)
COPD, *n* (%)	4 (7.4)	1 (2.0)
Congestive heart failure, *n* (%)	11 (20.4)	9 (18.0)
Atrial fibrillation, *n* (%)	15 (27.8)	14 (28.0)
Hemoglobin, g/L	142.3 ± 12.7	139.7 ± 11.8
Creatinine, µmol/L	103.4 ± 92.7	88.3 ± 14.4
STS risk score, %	1.6 ± 2.4	1.4 ± 2.2
Echocardiographic characteristics		
Effective regurgitant orifice area, mm^2^	0.54 ± 0.27	0.61 ± 0.38
Preoperative mean mitral gradient, mmHg	2.3 ± 1.4	2.1 ± 1.0
Preoperative left ventricle ejection fraction, %	60.9 ± 5.5	61.4 ± 5.4
Operative characteristics		
Annuloplasty size, mm	33 ± 3	33 ± 4
Type of resection, *n*		
Triangular	33	2
Quadrangular	12	1
Quadrangular with sliding plasty	5	1
Not described	4	0
Concomitant procedures, *n* (%)		
Maze procedure	10 (18.5)	5 (10.0)
Tricuspid annuloplasty	4 (0.7)	4 (2.0)

*Note*: Continuous variables presented as mean ± *SD*.

Abbreviations: BMI, body mass index; BSA, body surface area; COPD, chronic obstructive pulmonary disease; NYHA, New York Heart Association.

### 3.2 Summary of reverse remodeling process

**Figure 1:**
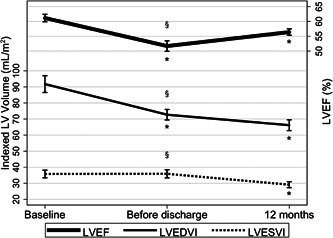
Remodeling trends in total cohort after mitral repair. § Represents *p* < .05 for ANOVA and *represents *p* < .05 when compared with the previous time point. ANOVA, analysis of variance; LV, left ventricular; LVEDVI, left ventricular end‐diastolic volume index; LVEF, left ventricular ejection fraction; LVESVI, left ventricular end‐systolic volume index

The overall trends in the change of LVESVI, LVEDVI, and LVEF amongst all patients included in these analyses are summarized in Figure 1 and detailed further in Table S2. The reverse remodeling in the immediate postoperative phase was characterized by a significant decrease in LVEDVI (72.7 ± 16.0 ml/m^2^ vs. 91.8 ± 22.6 ml/m^2^; *p* < .0001) with no change in LVESVI (35.9 ± 12.5 ml/m^2^ vs. 35.8 ± 10.3 ml/m^2^; *p* = .32); as such, LVEF also decreased (51.7 ± 8.7% vs. 61.1 ± 5.4%; *p* < .0001). Between discharge and the 12 months postoperative follow‐up, there was a further decrease in LVEDVI (66.2 ± 16.2 ml/m^2^ vs. 72.7 ± 16.0 ml/m^2^; *p* < .0018), as well as a significant decrease in LVESVI (29.1 ± 8.9 ml/m^2^ vs. 35.9 ± 12.5 ml/m^2^; *p* < .0001), resulting in an increase in LVEF (56.4 ± 5.1% vs. 51.7 ± 8.7%; *p* < .0001).

### 3.3 Predictors of early LV reverse remodeling

The results of the multivariate analysis for predicting early indices of LV reverse remodeling including LVESVI, LVEDVI, and LVEF amongst all investigated patients are presented in Table 2, and the results of the corresponding univariate analysis are available in Table S3. Early LV reverse remodeling before discharge was independently predicted by preoperative LV size. More specifically, both LVESVI and LVEDVI at discharge were predicted uniquely by their corresponding preoperative values (*p* < .001 for both associations), while discharge LVEF was associated with preoperative LVESVI (*p* < .001).

**Table 2:** Multivariate analysis for discharge LVESVI, LVEDVI, and LVEF

	Discharge LVESVI			Discharge LVEDVI			Discharge LVEF		
	Coef.	95% CI	*p* > *t*	Coef.	95% CI	*p* > *t*	Coef.	95% CI	*p* > *t*
Gender	−2.14	(−9.04, 4.77)	.53	−4.24	(−13.16, 4.68)	.35			
Creatinine	0.012	(−0.018, 0.043)	.43						
Atrial fibrillation							−3.79	(−8.20, 0.62)	.09
Preoperative LVEF	0.09	(−0.44, 0.62)	.73				0.21	(−0.21, 0.63)	.32
Preoperative LVESVI	0.84	(0.51, 1.17)	<.001				−0.54	(−0.79, −0.30)	<.001
Preoperative LVEDVI				0.37	(0.21, 0.54)	<.001			
Preoperative mitral septal‐apical dimension	−0.09	(−0.64, 0.46)	.74	−0.06	(−0.78, 0.67)	.88	0.18	(−0.24, 0.60)	.40
Preoperative RVD	0.08	(−0.52, 0.68)	.79	0.33	(−0.46, 1.11)	.41	0.22	(−0.23, 0.68)	.33
Preoperative tricuspid diameter	−0.06	(−0.70, 0.57)	.84	0.09	(−0.68, 0.85)	.82	0.06	(−0.42, 0.54)	.82
Annuloplasty ring size	0.83	(−0.04, 1.70)	.06	0.82	(−0.29, 1.94)	.15	−0.47	(−1.12, 0.17)	.15

Abbreviations: CI, confidence interval; Coef, coefficient; LVEDVI, left ventricular end‐diastolic volume index; LVEF, left ventricular ejection fraction; LVESVI, left ventricular end‐systolic volume index; RVD, right ventricular dimension.

### 3.4 Predictors of change in LV reverse remodeling parameters from predischarge to 12 months postoperatively

The independent predictive factors of the change in LVESVI, LVEDVI, and LVEF from predischarge to 12 months postoperative are presented in Table 3. The results of the corresponding univariate analysis are available in Table S4. The change in LVESVI was significantly associated with the size of the mitral ring used (*p* < .05) and the mean pressure gradient across the mitral valve at discharge (*p* < .05). Similarly, the change in LVEDVI was also predicted by the predischarge measurement of the mean mitral valve gradient (*p* < .05). Finally, the change in LVEF was significantly predicted by the size of the mitral ring implanted and preoperative LVESVI (*p* < .01 for both associations).

**Table 3:** Multivariate analysis for change in LVESVI, LVEDVI, and LVEF from predischarge to 12 months postoperatively

	∆ LVESVI			∆ LVEDVI			∆ LVEF		
	Coef.	95% CI	*p > t*	Coef.	95% CI	*p > t*	Coef.	95% CI	*p > t*
Smoking				−6.51	(−13.61, 0.59)	.072			
Preoperative LVESVI							0.24	(0.09, 0.38)	.002
Discharge mean mitral valve gradient	1.95	(0.02, 3.88)	.048	3.08	(0.42, 5.74)	.024	−1.26	(−2.68, 0.16)	.082
Perfusion time							−0.04	(−0.10, 0.01)	.13
Annuloplasty ring size	−0.86	(−1.64, −0.08)	.032				0.75	(0.22, 1.29)	.007

Abbreviations: CI, confidence interval; Coef, coefficient; LVEDVI, left ventricular end‐diastolic volume index; LVEF, left ventricular ejection fraction; LVESVI, left ventricular end‐systolic volume index.

## 4 DISCUSSION

The present sub‐analysis of the CAMRA CardioLink‐2 study significantly contributes to the broader understanding of postmitral repair reverse remodeling and its predictive factors through three central findings. First, the findings reveal a signification association between preoperative LV dimensions and both early as well as midterm remodeling following mitral valve repair. Second, favorable reverse remodeling from predischarge to 12 months postoperatively was independently predicted by larger mitral annulus size intraoperatively and lower mean pressure gradient across the mitral valve at discharge. Finally, mitral valve repair technique and coaptation height demonstrated no significant correlation with the reverse remodeling process, confirming the relative equivalence of the two strategies as previously reported.^25^

The current findings corroborate and extend on the literature to date that has underscored the importance of surgically addressing MR before the development of significant cardiac remodeling. Specifically, earlier studies have suggested that treating patients with mitral valve repair before significant increases in LV diameters or mass, as well as before decreases in LVEF, may enable for more effective reverse remodeling and recovery of LV mass, geometry and function.^7^, ^9^, ^10^, ^13^, ^17^, ^18^, ^19^, ^20^, ^21^, ^22^, ^27^, ^28^, ^29^ For example, in a retrospective analysis of 303 patients with leaflet prolapse that were treated with mitral valve repair, Tribouilloy et al.^22^ found a significant decrease in LVEF following surgery, and identified the preoperative cut‐offs of <64% for LVEF and ≥37 mm for LV end‐systolic diameter (LVESD) as the most fruitful for predicting LVEF < 50% at approximately 11 months postoperatively. Notably, the authors highlighted that the LV dysfunction frequency increased amongst patients meeting both of the aforementioned criteria, compared to those meeting just one. The importance of not only focusing on LV function in the decision making process for timing of surgery is of particular relevance, despite some touting LVEF as the most useful indicator of LV systolic function in MR patients.^30^ Indeed, because of the volume overload and reduced afterload in persons with severe MR, preoperative LVEF values may be falsely inflated and may therefore conceal LV injury or dysfunction.^2^, ^13^, ^31^, ^32^ Previous histological examination has linked this MR‐related LV contractile dysfunction to myofibrillar damage secondary to oxidative stress even amongst patients with “normal” LVEF values.^2^

In the present study, the change in indices of reverse remodeling were independently predicted by mitral annuloplasty size and the mean pressure gradient across the valve at discharge. These data suggest that optimizing mitral valve hemodynamics with effective surgical repair and appropriate medical therapy in the acute period following surgery may encourage myocardial reverse remodeling. With regard to annuloplasty size, a meta‐analysis showed that resection repair was associated with smaller annuloplasty size.^33^ Although in our study, the annuloplasty size was identical between the groups, additional attention may be warranted for resection repair. Previously reported predictors for postoperative reverse remodeling following mitral valve repair include echocardiographic parameters,^7^, ^10^, ^13^, ^17^, ^18^, ^19^, ^20^, ^21^, ^22^, ^23^, ^27^, ^28^, ^29^, ^34^ clinical and patient factors,^13^, ^17^, ^19^, ^20^, ^21^, ^23^, ^24^ laboratory measurements,^20^ and operative variables.^8^, ^21^, ^23^ There is, however, mounting evidence supporting the use of advanced imaging techniques like magnetic resonance imaging, 3‐dimensional echocardiography, and speckle tracking,^9^, ^13^, ^28^ to detect occult myocardial dysfunction and thereby predict postoperative reverse remodeling capacity in patients with MR.

The current sub‐analyses have several limitations that must be acknowledged. First, this is a post hoc sub‐analysis and the findings should be considered exploratory. Second, limited available 3‐dimensional echocardiography and speckle tracking data prevented analysis of these parameters in predicting postoperative LV reverse remodeling. Third, the applicability of these findings to all patients with primary, degenerative MR may be limited given the exclusive inclusion of patients with posterior mitral valve prolapse and the recruitment of highly experienced mitral valve surgeons. Also, our study cohort may consist of MR patients relative in early phase by excluding low LVEF, for example. This may limit generalizability of our study findings. Fourth, the 12 months postoperative follow‐up did not allow us to determine if a lack of reverse remodeling postmitral valve repair may contribute to quality of life outcomes that may be more critical from the perspective of patients. Future studies should build on these findings through examining the long‐term associations of cardiac reverse remodeling and clinical outcomes including heart failure recurrence and cardiovascular death. Despite these limitations, the strengths of this sub‐analysis are the randomized and prospective nature of the data as well as the high internal validity given the rather specific population investigated and the blinding of all outcome assessors to repair strategy and image timing.

## 5 CONCLUSIONS

The present findings from the CAMRA CardioLink‐2 randomized trial suggest that early surgery before LV dilatation and dysfunction, as well as the use of intraoperative and postoperative management techniques to optimize mitral valve hemodynamics acutely following surgery, may improve the course of reverse remodeling following mitral valve repair. This investigation also reinforces the relative equivalence of leaflet resection and leaflet preservation technique in inducing postoperative reverse remodeling following mitral valve repair.

## CONFLICTS OF INTEREST

C. David Mazer reports advisory board honoraria from Amgen, AstraZeneca, Boehringer Ingelheim, and Octapharma. Howard Leong‐Poi hold the Brazilian Ball Chair in Cardiology and reports receiving honoraria for speaking engagements from Lantheus Medical Imaging and Janssen. Subodh Verma holds a Tier 1 Canada Research Chair in Cardiovascular Surgery; and reports receiving research grants and/or speaking honoraria from Amarin, Amgen, AstraZeneca, Bayer, Boehringer Ingelheim, Bristol‐Myers Squibb, Eli Lilly, EOCI Pharmacomm Ltd, HLS Therapeutics, Janssen, Merck, Novartis, Novo Nordisk, Pfizer, PhaseBio, Sanofi, Sun Pharmaceuticals, and the Toronto Knowledge Translation Working Group. He is the President of the Canadian Medical and Surgical Knowledge Translation Research Group, a federally incorporated not‐for‐profit physician organization. KAC is listed as an inventor on a patent application by Boehringer Ingelheim on the use of dipeptidyl peptidase‐4 inhibitors in heart failure; and reports receiving research grants to his institution from AstraZeneca, Servier and Boehringer Ingelheim; support for travel to scientific meetings from Boehringer Ingelheim and honoraria for speaking engagements and ad hoc participation in advisory boards from Servier, Merck, Eli Lilly, AstraZeneca, Boehringer Ingelheim, Ferring, Novo Nordisk, Novartis and Janssen. All other authors report no relevant conflicts of interest.

## Supporting information

Supporting information.

## Data Availability

Author elects to not share data.
