# Supplementary material for: Stage‐based approach to predict left ventricular reverse remodeling after mitral repair
Source: Clin Cardiol. 2022 Jun 24;45(9):921–7. doi: 10.1002/clc.23879 (PMC9451668; doi:10.1002/clc.23879)
Supplement: Supplementary file 1 — Supporting information. [file CLC-45-921-s001.docx]

*Supplementary Table 1. Baseline Echocardiographic findings*

|  | **Baseline** | | |
| --- | --- | --- | --- |
|  | Mean | SD | |
| **LVEDD (mm)** | 54.5 | ± | 5.6 |
| **LVESD (mm)** | 35.7 | ± | 5.9 |
| **LVEDVI (ml/m^2^)** | 91.8 | ± | 22.6 |
| **LVESVI (ml/m^2^)** | 35.8 | ± | 10.3 |
| **LVEF (%)** | 61.1 | ± | 5.4 |
| **LVOT VTI (cm)** | 17.0 | ± | 4.1 |
| **Indexed LA volume (mL/m^2^)** | 55.2 | ± | 21.4 |
| **Mitral Septal-Apical Dimension (mm)** | 40.8 | ± | 5.2 |
| **Mitral Bicommissural Dimension (mm)** | 43.8 | ± | 5.3 |
| **RVD (mm)** | 41.2 | ± | 5.8 |
| **TAPSE (mm)** | 23.5 | ± | 4.0 |
| **TR** |  |  |  |
| **None, n (%)** | 9 |  | (9.7) |
| **Mild, n (%)** | 79 |  | (85.0) |
| **Moderate, n (%)** | 5 |  | (5.4) |
| **ePAP (mmHg)** | 36.3 | ± | 14.7 |

Continuous variables presented as mean ± SD.

ePAP, estimated pulmonary artery pressure; LA, left atrium; LVEDD, left ventricular end-diastolic diamter; LVEDVI, left ventricular end-diastolic volume index; LVEF, left ventricular ejection fraction; LVESD, left ventricular end-systolic diameter; LVESVI, left ventricular end-systolic volume index; LVOT VTI, left ventricular outflow tract velocity time integral; RVD, right ventricular dimension; TAPSE, Tricuspid annular plane systolic excursion; TR, tricuspid regurgitation

*Supplementary Table 2. Remodeling trends in total cohort after mitral repair*

|  | Baseline | | | Before discharge | | | vs Baseline | 12 months | | | vs Before discharge | ANOVA |
| --- | --- | --- | --- | --- | --- | --- | --- | --- | --- | --- | --- | --- |
|  | Mean | SD | | Mean | SD | | p | Mean | SD | | p | p |
| LVEDVI (mL/m^2^) | 91.8 | ± | 22.6 | 72.7 | ± | 16.0 | <0.0001 | 66.2 | ± | 16.2 | 0.0018 | <0.0001 |
| LVESVI (mL/m^2^) | 35.8 | ± | 10.3 | 35.9 | ± | 12.5 | 0.32 | 29.1 | ± | 8.9 | <0.0001 | <0.0001 |
| LVEF (%) | 61.1 | ± | 5.4 | 51.7 | ± | 8.7 | <0.0001 | 56.4 | ± | 5.1 | <0.0001 | <0.0001 |

ANOVA, analysis of variance; LVEDVI, left ventricular end-diastolic volume index; LVEF, left ventricular ejection fraction; LVESVI, left ventricular end-systolic volume index

*Supplementary Table 3. Univariate analysis for pre-discharge LVESVI, LVEDVI, and LVEF.*

|  | Pre-discharge LVESVI | | Pre-discharge LVEDVI | | Pre-discharge LVEF | |
| --- | --- | --- | --- | --- | --- | --- |
|  | **Coef.** | **95% CI** | **Coef.** | **95% CI** | **Coef.** | **95% CI** |
| Age | 0.03 | (-0.24, 0.30) | 0.03 | (-0.31, 0.37) | 0.01 | (-0.17, 0.20) |
| Gender | -9.80 | (-16.16, -3.44) | -14.61 | (-22.63, -6.59) | 3.34 | (-1.27, 7.95) |
| Hypertension | -3.40 | (-8.47, 1.67) | -2.61 | (-9.16, 3.94) | 2.60 | (-0.92, 6.11) |
| Hypercholesterolemia | 0.94 | (-4.23, 6.12) | 2.11 | (-4.52, 8.75) | 0.53 | (-3.07, 4.12) |
| Hemoglobin | 0.03 | (-0.18, 0.23) | 0.02 | (-0.25, 0.28) | -0.03 | (-0.18, 0.11) |
| Creatinine | 0.04 | (0.00, 0.074) | 0.04 | (-0.00, 0.09) | -0.02 | (-0.05, 0.01) |
| Smoking | 2.04 | (-3.23, 7.31) | 3.09 | (-3.71, 9.90) | -0.56 | (-4.21, 3.10) |
| Heart Rate | 0.14 | (-0.05, 0.33) | 0.02 | (-0.22, 0.27) | -0.13 | (-0.25, -0.01) |
| Atrial Fibrillation | 3.60 | (-2.16, 9.35) | 1.29 | (-6.16, 8.75) | -4.56 | (-8.45, -0.67) |
| Preoperative LVEF | -0.72 | (-1.27, -0.17) | -0.44 | (-1.15, 0.27) | 0.70 | (0.34, 1.06) |
| Preoperative LVEDVI | 0.40 | (0.29, 0.50) | 0.47 | (0.34, 0.61) | -0.20 | (-0.28, -0.11) |
| Preoperative LVESVI | 0.92 | (0.71, 1.14) | 0.99 | (0.69, 1.29) | -0.55 | (-0.71, -0.38) |
| Preoperative LVOT VTI | -0.68 | (-1.52, 0.16) | -0.45 | (-1.50, 0.60) | 0.62 | (0.05, 1.20) |
| Preoperative LA Volume Index | 0.14 | (-0.00, 0.28) | 0.19 | (0.03, 0.36) | -0.05 | (-0.15, 0.05) |
| Preoperative Mitral Septal-Apical Dimension | 0.83 | (0.22, 1.44) | 0.99 | (0.22, 1.75) | -0.43 | (-0.86, -0.008) |
| Preoperative Mitral Bicommissural Dimension | 0.53 | (-0.10, 1.15) | 0.71 | (-0.06, 1.49) | -0.22 | (-0.65, 0.22) |
| Preoperative RVD | 1.03 | (0.58, 1.47) | 1.29 | (0.73, 1.85) | -0.49 | (-0.82, -0.16) |
| Baseline TAPSE | 0.01 | (-1.01, 1.02) | 0.64 | (-0.53, 1.81) | 0.40 | (-0.34, 1.14) |
| Preoperative Tricuspid Diameter | 0.82 | (0.32, 1.33) | 0.89 | (0.25, 1.53) | -0.48 | (-0.84, -0.12) |
| Preoperative ePAP | 0.17 | (-0.07, 0.42) | 0.25 | (-0.04, 0.55) | -0.02 | (-0.21, 0.16) |
| Clamp time | 0.01 | (-0.09, 0.10) | -0.01 | (-0.13, 0.12) | -0.01 | (-0.08, 0.06) |
| Perfusion Time | 0.00 | (-0.08, 0.08) | -0.03 | (-0.13, 0.08) | -0.02 | (-0.07, 0.04) |
| Concomitant TAP | 0.25 | (-12.48, 12.98) | 10.27 | (-5.94, 26.48) | 4.79 | (-4.06, 13.63) |
| Randomized Allocation | 0.98 | (-4.12, 6.09) | 2.23 | (-4.33, 8.78) | 1.07 | (-2.48, 4.62) |
| Use of Resection Repair | -2.56 | (-7.67, 2.55) | -3.35 | (-9.91, 3.21) | 0.90 | (-2.67, 4.47) |
| Neochord Use | 0.09 | (-5.07, 5.25) | 1.03 | (-5.59, 7.66) | 0.80 | (-2.79, 4.39) |
| Annuloplasty Ring Size | 1.25 | (0.50, 2.00) | 1.48 | (0.51, 2.45) | -0.67 | (-1.21, -0.12) |

Coef, coefficient; CI, confidence interval; ePAP, estimated pulmonary artery pressure; LA, left atrium; LVEDVI, left ventricular end-diastolic volume index; LVEF, left ventricular ejection fraction; LVESVI, left ventricular end-systolic volume index; LVOT VTI, left ventricular outflow tract velocity time integral; RVD, right ventricular dimension; TAP, tricuspid annuloplasty; TAPSE, Tricuspid annular plane systolic excursion

*Supplementary Table 4. Univariate analysis for change in LVESVI, LVEDVI, and LVEF from pre-discharge to 12 months postoperatively.*

|  | dLVESVI1 | | dLVEDVI1 | | dLVEF1 | |
| --- | --- | --- | --- | --- | --- | --- |
|  | **Coef.** | **95% CI** | **Coef.** | **95% CI** | **Coef.** | **95% CI** |
| Age | -0.04 | (-0.30, 0.21) | -0.13 | (-0.52, 0.25) | -0.03 | (-0.18, 0.12) |
| Gender | 4.33 | (-1.71, 10.36) | 3.69 | (-5.44, 12.82) | -2.40 | (-6.00, 1.20) |
| Hypertension | 4.32 | (-0.35, 8.98) | 5.60 | (-1.43, 12.63) | -2.27 | (-5.05, 0.50) |
| Hypercholesterolemia | -2.55 | (-7.36, 2.26) | -4.57 | (-11.77, 2.62) | -0.19 | (-3.05, 2.67) |
| Hemoglobin | 0.01 | (-0.19, 0.20) | -0.04 | (-0.33, 0.26) | -0.02 | (-0.13, 0.10) |
| Creatinine | 0.01 | (-0.02, 0.05) | 0.03 | (-0.02, 0.08) | 0.00 | (-0.02, 0.02) |
| Smoking | -4.07 | (-8.96, 0.81) | -7.28 | (-14.52, -0.025) | 0.20 | (-2.74, 3.13) |
| Postoperative Beta-Blocker | -0.53 | (-5.31, 4.24) | 2.65 | (-4.49, 9.80) | 2.06 | (-0.74, 4.86) |
| Postoperative ACE inhibitor | 1.38 | (-4.88, 7.63) | 1.09 | (-8.30, 10.49) | -0.62 | (-4.26, 3.01) |
| Heart Rate | -0.03 | (-0.21, 0.16) | 0.02 | (-0.27, 0.30) | 0.04 | (-0.06, 0.13) |
| Atrial Fibrillation | 0.41 | (-5.22, 6.04) | 0.04 | (-8.41, 8.48) | 0.42 | (-2.87, 3.71) |
| Preoperative LVEF | 0.05 | (-0.50, 0.59) | -0.00 | (-0.80, 0.79) | -0.01 | (-0.33, 0.30) |
| Preoperative LVEDVI | -0.13 | (-0.26, 0.01) | 0.06 | (-0.13, 0.26) | 0.15 | (0.09, 0.22) |
| Preoperative LVESVI | -0.25 | (-0.55, 0.04) | 0.12 | (-0.32, 0.56) | 0.29 | (0.14, 0.45) |
| Preoperative LVOT VTI | 0.02 | (-0.77, 0.80) | 0.07 | (-1.04, 1.19) | 0.12 | (-0.34, 0.58) |
| Preoperative LA volume Index | -0.03 | (-0.16, 0.10) | -0.05 | (-0.23, 0.14) | -0.01 | (-0.09, 0.07) |
| Preoperative Mitral Septal-Apical Dimension | -0.44 | (-1.09, 0.22) | -0.45 | (-1.42, 0.52) | 0.15 | (-0.24, 0.54) |
| Preoperative Mitral Bicommissural Dimension | -0.52 | (-1.14, 0.11) | -0.69 | (-1.61, 0.24) | 0.17 | (-0.20, 0.54) |
| Preoperative RVD | -0.32 | (-0.78, 0.13) | -0.16 | (-0.83, 0.51) | 0.18 | (-0.10, 0.45) |
| Baseline TAPSE | -0.18 | (-1.13, 0.77) | -0.39 | (-1.74, 0.96) | 0.07 | (-0.49, 0.63) |
| Preoperative Tricuspid Diameter | -0.34 | (-0.84, 0.16) | -0.39 | (-1.13, 0.35) | 0.12 | (-0.18, 0.41) |
| Preoperative ePAP | -0.19 | (-0.40, 0.02) | -0.20 | (-0.49, 0.10) | 0.06 | (-0.07, 0.20) |
| Discharge Peak Gradient | 0.67 | (-0.11, 1.45) | 0.37 | (-0.82, 1.56) | -0.46 | (-0.92, 0.01) |
| Discharge Mean Gradient | 2.83 | (1.09, 4.56) | 3.16 | (0.48, 5.84) | -1.41 | (-2.46, -0.36) |
| Residual MR ≧mild at discharge | -1.68 | (-7.40, 4.04) | 0.23 | (-8.36, 8.83) | 2.79 | (-0.50, 6.09) |
| TR ≧ mild at discharge | 1.08 | (-5.03, 7.18) | 2.11 | (-7.05, 11.26) | 0.13 | (-3.51, 3.76) |
| Intraoperative Mitral Coaptation height | 0.13 | (-0.74, 0.99) | 0.12 | (-1.18, 1.41) | -0.28 | (-0.80, 0.24) |
| Clamp Time | 0.06 | (-0.04, 0.16) | 0.04 | (-0.10, 0.19) | -0.06 | (-0.11, -0.00) |
| Perfusion Time | 0.07 | (-0.00, 0.15) | 0.08 | (-0.03, 0.19) | -0.05 | (-0.09, -0.006) |
| Concomitant TAP | 3.62 | (-7.65, 14.88) | 1.84 | (-15.01, 18.77) | -3.26 | (-9.95, 3.43) |
| Use of resection repair | 2.14 | (-2.62, 6.91) | -0.86 | (-8.04, 6.33) | -2.38 | (-5.17, 0.42) |
| Neochord use | -0.83 | (-5.63, 3.97) | 1.44 | (-5.76, 8.64) | 0.95 | (-1.89, 3.79) |
| Annuloplasty Ring Size | -1.22 | (-1.93, -0.52) | -1.01 | (-2.12, 0.11) | 0.85 | (0.44, 1.26) |

Coef, coefficient; CI, confidence interval; ePAP, estimated pulmonary artery pressure; LA, left atrium; LVEDVI, left ventricular end-diastolic volume index; LVEF, left ventricular ejection fraction; LVESVI, left ventricular end-systolic volume index; LVOT VTI, left ventricular outflow tract velocity time integral; MR, mitral regurgitation; RVD, right ventricular dimension; TAP, tricuspid annuloplasty; TAPSE, Tricuspid annular plane systolic excursion; TR, tricuspid regurgitation
